# Supplementary material for: MetaRibo-Seq measures translation in microbiomes
Source: Nat Commun. 2020 Jun 29;11:3268. doi: 10.1038/s41467-020-17081-z (PMC7324362; doi:10.1038/s41467-020-17081-z)
Supplement: Supplementary file 10 — Supplementary Data 7 [file 41467_2020_17081_MOESM10_ESM.zip › File2/Confidence_High_Taxonomy/433905_out.krona.html]

Javascript must be enabled to view this page.

members
magnitude
magnitudeUnassigned
count
unassigned
taxon
rank

433905\_out

8

superkingdom
2
8

phylum
8
1224

1236
8
class

order
8
91347

543
8
family

genus
8
561


SRS017191\_contig\_number\_20052SRS017521\_contig\_number\_51148SRS049402\_contig\_number\_16994SRS075947\_contig\_number\_15993SRS076876\_contig\_number\_966SRS144362\_contig\_number\_22046SRS146888\_contig\_number\_15618SRS147022\_contig\_number\_13928
species
562
8
